# High capacity and stable all-solid-state Li ion battery using SnO_2_-embedded nanoporous carbon

**DOI:** 10.1038/s41598-018-27040-w

**Published:** 2018-06-08

**Authors:** Hiroo Notohara, Koki Urita, Hideyuki Yamamura, Isamu Moriguchi

**Affiliations:** 1 Graduate School of Engineering, Nagasaki University, 1-14 Bunkyo-machi, Nagasaki-shi, Nagasaki 852-8521 Japan; 2 TOYOTA Motor Corporation, 1200 Mishuku, Susono-shi, Shizuoka, 410-1193 Japan

## Abstract

Extensive research efforts are devoted to development of high performance all-solid-state lithium ion batteries owing to their potential in not only improving safety but also achieving high stability and high capacity. However, conventional approaches based on a fabrication of highly dense electrode and solid electrolyte layers and their close contact interface is not always applicable to high capacity alloy- and/or conversion-based active materials such as SnO_2_ accompanied with large volume change in charging-discharging. The present work demonstrates that SnO_2_-embedded nanoporous carbons without solid electrolyte inside the nanopores are a promising candidate for high capacity and stable anode material of all-solid-state battery, in which the volume change reactions are restricted in the nanopores to keep the constant electrode volume. A prototype all-solid-state full cell consisting of the SnO_2_-based anode and a LiNi_1/3_Co_1_/_3_Mn_1/3_O_2_-based cathode shows a good performance of 2040 Wh/kg at 268.6 W/kg based on the anode material weight.

## Introduction

All-solid-state lithium ion batteries (ASS−LIBs) with nonflammable solid electrolyte have attracted growing interest because of the safety precaution required for widespread use of Li ion batteries (LIBs) in power-grid applications, electric vehicles and so on^1,2^. Although the low ionic conductivity of solid electrolyte (SE) has limited so far the development of ASS−LIB to a thin film-type cell^3–5^, the recent discovery of highly ionic conductive SE such as sulfide-based compounds, of which conductivity was over 10^−3^ S cm^−1^ (refs^6–10^), prompt us to develop a bulk-type cell composed of active materials and SE powders. This is expected to lead high capacity ASS-LIBs instead of LIBs using organic liquid electrolytes (OLE−LIBs).

Conventional researches on ASS−LIBs have focused on fabrication of highly dense electrode layer and SE layer as well as their close contact interface to yield enough Li ion conducting paths^11–14^, which is quite different from OLE−LIBs. However, large capacity active materials such as Si, Sn, P, SnO_2_ and so on, which are accompanied with large volume change in their Li−alloying/dealloying and metal oxide to metal conversion reactions, are difficult to apply to the present ASS−LIB system. The close contact at the SE/electrode interface and densely packing in the electrode are not maintained for such active materials due to the formation of crack and void with the volume change during charge-discharge cycling. It was reported that an increase in compressive pressure of ASS-cell using silicon and Li electrodes to keep the interface contact improved the cycle performance, but the capacity was decreased due to the limitation of reactions^15,16^.

Here, we report on the first attempt of applying active material-embedded porous carbon electrode materials to ASS−LIBs. The novel strategy of this study is as follows; (1) the porous carbon framework is used as a three-dimensional electron conducting path in the electrode, (2) the carbon nanopores play an important role of a buffer space for the volume change during charge−discharge reactions to keep the constant electrode volume and then a stable contact of SE/electrode interface. Porous materials as a battery electrode are considered to have disadvantage of low volumetric capacity, but this is not the case for the present study because the pore space provided in advance is compensated by the inevitable volume expansion of lithiated active materials. Here we found out that a SnO_2_−embedded nanoporous carbon fulfills their functions as the active electrode material in ASS system and surprisingly shows high capacity and good cycle stability superior to those in OLE system. One more fascinating discovery of this study is that Li ion conduction occurs successfully in the carbon nanospace without SE beyond expectation. The approach is applicable to other alloy- and conversion-based active materials with large volume change in principle, thus the present study will open the door for the development of high performance ASS-LIBs with high capacity and high stability.

## Results

### Preparation and characterization of SnO_2_–embedded nanoporous carbons

**Figure 1:**
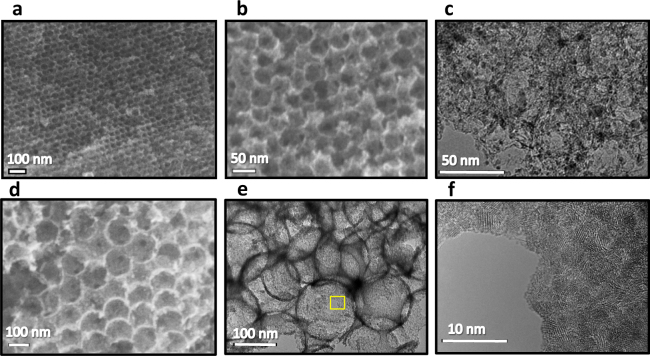
Porous composite structure of SnO_2_/C*X*[*Y*]. (**a,b**) SEM images of SnO_2_/C45[73]; (**b**) is an enlarge image. (**c**) TEM image of SnO_2_/C45[73]. (**d**) SEM image of SnO_2_/C140[75]. (**e**) TEM image of SnO_2_/C140[75]. (**f**) Enlarged image of the yellow frame area in **e**.

Embedding of SnO_2_ nanocrystallites into nanopores of porous carbons was carried out by introducing SnCl_2_ vapor into the carbon nanopores, a subsequent hydrolysis and dryness according to the previous report^17,18^. A porous carbon with an average pore diameter of 45 or 140 nm, which was obtained by a silica opal template process^19^, was used here for the above syntheses. In the following, the porous carbon and the SnO_2_-embedded carbon nanocomposites are denoted as C*X* and SnO_2_/C*X*[*Y*], where *X* and *Y* indicate the average pore diameter of porous carbon and the loading amount of SnO_2_, respectively. Powder X-ray diffraction (XRD) measurements confirmed that SnO_2_ nanocrystallites with the primary crystallite size around 3 nm, which was estimated from the full width at half-maximum of (110) XRD peak using the Scherrer equation, were produced in the composite samples (Supplementary Fig. 1). It was observed by scanning electron microscopy (SEM) and transmission electron microscopy (TEM) that SnO_2_ nanocrystallites with the size around 3 nm, which was consistent with the primary crystallite size estimated from XRD, were deposited preferentially in the carbon nanopores (Fig. 1). As shown in Fig. 1f, the SnO_2_ nanocrystallites were closely accumulated on the surface of carbon pore wall for the high SnO_2_-loading sample of SnO_2_/C140[75]. Table 1 shows structural parameters of SnO_2_ contents, specific surface areas (*S*_a_) and specific pore volumes (*V*_p_) of samples, as well as weighted average values of *S*_a_ (*S*_a,w_) and *V*_p_ (*V*_p,w_) which are calculated under the assumption of a simple mixing of C*X* and SnO_2_ nanocrystallites with the primary crystallite size. The *S*_a_ and *V*_p_ values were decreased with increasing the loading amount of SnO_2_ and they were much smaller than the weighted average values. In addition, the pore size distribution was shifted toward smaller pore size range with the increase in SnO_2_ loading amount (Supplementary Fig. 2). These results demonstrated almost perfect embedding of SnO_2_ nanocrystallites in carbon nanopores of C*X*.

**Table 1:** Structural parameters of SnO_2_/C*X*[*Y*].

Sample	*Y* (wt%)	*S*_a_ (m^2^/g)	*S*_a,w_ (m^2^/g)	*V*_p_ (cm^3^/g)	*V*_p,w_ (cm^3^/g)
SnO_2_/C140[*Y*]	0 61 70	1073 326 279	1073 594 523	2.00 0.56 0.38	2.00 0.78 0.60
SnO_2_/C45[*Y*]	0 56 68 72	1093 548 223 220	1093 604 518 489	4.42 1.82 0.92 0.90	4.42 1.94 1.41 1.24

*Y*: SnO_2_ content, *S*_a_: Specific surface area, *V*_p_: specific pore volume, *S*_a,w_ and *V*_p,w_: calculated values of *S*_a_ and *V*_p_ under the assumption of a simple mixing of C*X* and SnO_2_.

### Charge-discharge properties of SnO_2_–embedded nanoporous carbons

**Figure 2:**
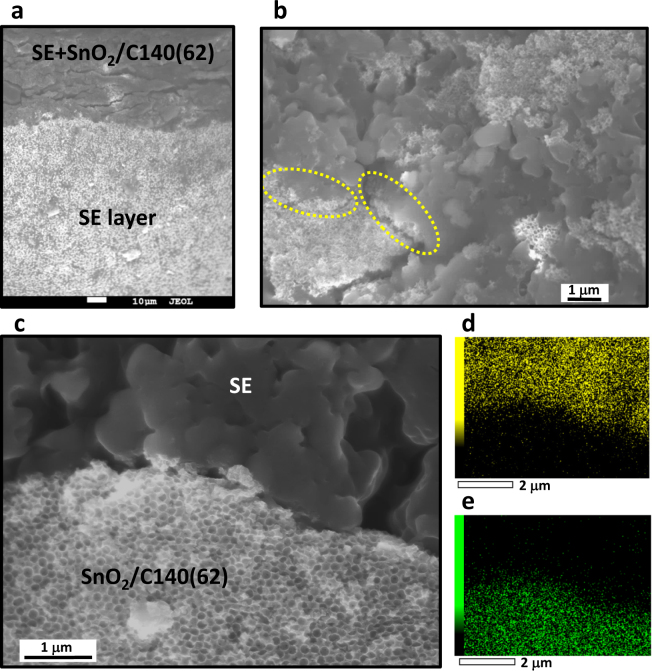
Cross-sectional morphology of SnO_2_/C140[62]-electrode layer and SE layer in disassembled ASS half-cell. (**a**) SEM image of the interface of these layers. (**b**) SEM image of the electrode layer part in **a**; the darker areas with smooth surface correspond to particulate and/or stretched SE. Rough and porous surface areas are of SnO_2_/C140[62] domains. The dotted yellow circle areas indicate representative SE-covered SnO_2_/C140[62] domains. (**c**) Enlarged SEM image of the interface between SE and SnO_2_/C140[62] large domain. (**d**,**e**) EDX elementary mapping of sulfur (**d**) and tin (**e**) in **c**. Here EDX data were taken on the large domain to avoid the additional signals behind domains.

The charge-discharge measurements were carried out on an ASS half-cell, which was composed of a working electrode (WE) layer of mixture of SnO_2_/CX[*Y*] and LiI-Li_2_S-P_2_S_5_ (SE), the SE layer and a Li foil (see experimental section). Figure 2a shows an SEM image of a cross section of SE layer and working electrode layer using SnO_2_/C140[62] of a disassembled ASS half-cell. Since the sample was peeled off from the current collector for the observation, there were some cracks in the WE layer. SE and SnO_2_/C140[62] domains were existed in the WE layer (Fig. 2b), and some of SnO_2_/C140[62] domains were covered by stretched SE, which was deformed from as-prepared SE particles during the milling and pressing processes for the cell construction (Supplementary Fig. 3). It was confirmed that the porous structure of SnO_2_/C140[62] was maintained without filling SE in nanopores even after the cell construction (Fig. 2c,d and e). Sight signals of S and Sn remained in the black area out of SnO_2_/C140[62] and SE domains in Fig. 2d,e, respectively, are due to inevitable background signals caused by continuous X-ray. No existence of the solid electrolyte inside the nanopores was also confirmed by STEM-EELS-EDX analysis (Supplementary Fig. 4).

**Figure 3:**
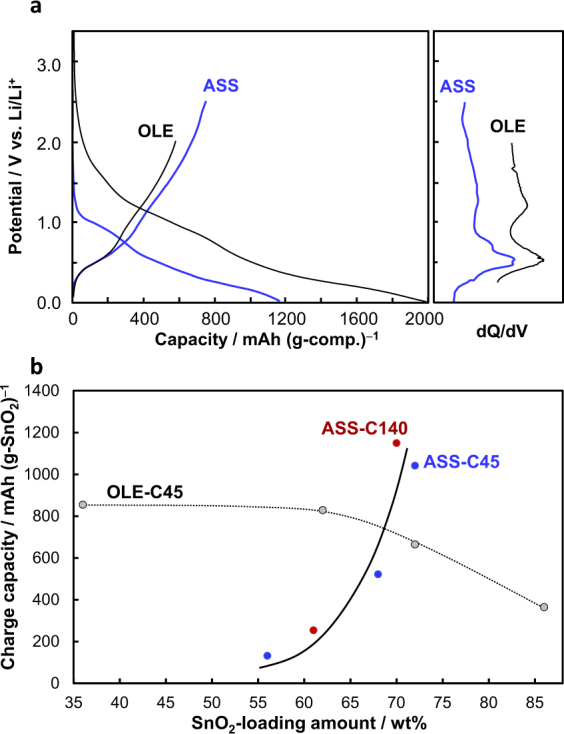
Initial charge-discharge properties of SnO_2_/C*X*[*Y*] versus Li/Li^+^ in all-solid state (ASS) and organic liquid electrolyte (OLE) systems. (**a**) Initial charge-discharge and d*Q*/d*V* curves of SnO_2_/C45[72]. (**b**) Initial charge capacity as a function of SnO_2_-loading amount of SnO_2_/C140[*Y*] (brown) and SnO_2_/C45[*Y*] (blue and gray).

In Fig. 3, the initial charge-discharge curves of SnO_2_/C45[72] are shown as a representative example and are compared with those measured in an organic liquid electrolyte (OLE) half-cell. The initial discharge process on the ASS system included irreversible capacity, but it was much smaller than that in the OLE due to the solid electrolyte interface formation. The ASS system surprisingly showed higher charge capacity than the OLE system and clear d*Q*/d*V* peaks of dealloying reaction of Li_x_Sn around 0.5 V and conversion reaction of Sn to SnO_y_ above 0.9 V, of which potentials are consistent with those in the OLE system and the previous reported. It was also confirmed that the potential of d*Q*/d*V* peaks was consistent with that of anodic peaks in CV curve (Supplementary Fig. 5). The initial charge capacity in the ASS system was 750 mAh/g based on the composite weight, which was corresponding to 1042 mAh/g based on SnO_2_ weight because the capacity due to C45 was negligibly small. The high capacity over the theoretical capacity of either Sn-Li alloying-dealloying reaction (781 mAh/g) or SnO_2_-Sn conversion reaction (711 mAh/g), indicating the contribution of both reactions on the charging-discharging. It can be concluded that the SnO_2_-embedded porous carbon functions successfully as a high capacity electrode active material in ASS system.

Although Li ions in OLE system can easily access to SnO_2_ in SnO_2_/CX[*Y*] composites via the penetration of liquid electrolyte into the carbon nanopore space, it is not the case for ASS system because the SE is located outside of carbon nanopores. Therefore, the higher capacity of SnO_2_/C45[72] in ASS system than that in OLE system suggests that any Li ions conduction path exists throughout the SnO_2_-embedded carbon nanospace. Considering this point, the effect of loading amount of SnO_2_ (*Y*) on the capacity was investigated on the ASS system. As shown in Fig. 3b, the capacity was increased with increasing the *Y* value and especially SnO_2_-loading at *Y* > 65 was effective to yield high capacity. The tendency is quite different from that in OLE system, which was previously reported that the capacity is almost constant at *Y* < 65 and it drops at *Y* > 65 (ref.^18^). Highly SnO_2_-loading at *Y* > 65 in carbon nanopores is necessary to have an effective Li-ion conducting path in the SnO_2_/CX[*Y*] composite from the outside SE for the ASS system, which may be formed through the phases such as Li_x_Sn, Sn and Li_x_O produced by the reaction of SnO_2_ nanoparticles with Li ions. A detailed investigation on the Li-ion conduction mechanism is now in progress.

### Prototype full cell performance

**Figure 4:**
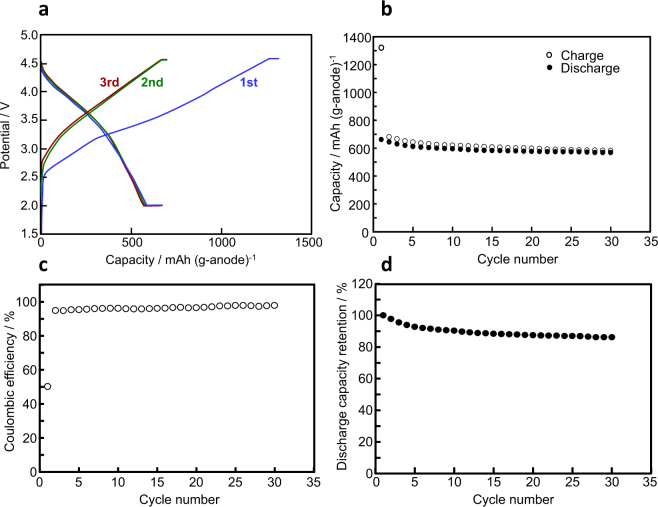
Performance of the prototype all-solid-state full cell. (**a**) Charge-discharge curves at 0.1 C and room temperature. (**b**) Cycle performance of capacity in the range of 2.0–4.5 V. (**c**) Coulombic efficiency versus cycle number. **(d)** Discharge capacity retention.

The charge-discharge properties during 30 cycling were investigated at room temperature on a prototype ASS full-cell using SnO_2_/C45[72] and LiNbO_3_-coated LiNi_1/3_Co_1_/_3_Mn_1/3_O_2_ as active anode and cathode materials, respectively (Fig. 4). The initial charge-discharge capacities based on anode material weight were comparable to those observed on the ASS half-cell taking into account that the anode/cathode capacity ratio was set at 1.2 for the cell assembly. The capacities after a few cycles were almost constant around 600 mAh/g-anode. The coulombic efficiency was above 95% after the 2^nd^ cycle and the discharge capacity retention was above 86% even after 30 cycles. The cycle performance in the ASS system was superior to that in the OLE system, of which capacity retention was 50% at the 30^th^ cycle as previously reported^18^. The average operating voltage of the ASS full-cell is 3.4 V, thus the specific energy density is considered to be 2040 Wh/kg at the specific power density of 268.6 W/kg based on the anode material weight. The prototype cell shows an even better performance than a liquid electrolyte-type cell.

## Discussion

In the present study, we disclosed that SnO_2_-embedded porous carbons are a promising candidate for stable and high capacity anode materials in ASS–LIB system. The high capacity is achieved on the samples with high SnO_2_-loading amount over 65 wt%, which is considered to be caused by a formation of effective Li ion conducting path in the SnO_2_-embedded carbon nanopores. Here we discuss the SnO_2_-loading amount-dependent property from simple calculations. When spherical nanoparticles of SnO_2_ with the diameter of 3 nm, which was confirmed by the TEM observation, are closely packed and form a single nanoparticulate layer on the surface of mesopores and macropores of original porous carbon, the coverage ratio of SnO_2_-related layer against the carbon pore surface is calculated to be 28–30% for the as-prepared SnO_2_/C45[72] and SnO_2_/C140[70] by using the specific mesopore and macropore surface areas of C45 (682 m^2^/g) and C140 (660 m^2^/g) determined by GCMC method. The coverage value is too low to carry Li ions via the loaded SnO_2_ throughout nanopores in SnO_2_/C*X*[*Y*] without filling SE inside unless all of SnO_2_ nanoparticles are located around the pore mouths contacted with outside SE. However, the coverage value is calculated to be 71–76% after the full reaction of SnO_2_ with Li ions, which is accompanied with the 2.12 time volume expansion for the conversion of SnO_2_ to Sn and Li_2_O as well as the 3.57 time volume expansion of Li_4.4_Sn against Sn, that is totally 4.05 time volume expansion based on SnO_2_ (Supplementary calculations). This means that effective Li ion conduction paths are formed in SnO_2_/C*X*[*Y*] from the outside SE probably through the phases such as Li_x_Sn, Sn and Li_x_O produced by the reaction of Li ions with SnO_2_ nanoparticles, thus the highly SnO_2_-loading is necessary to yield high capacity.

In summary, the use of the SnO_2_-embedded porous carbons was demonstrated as an effective strategy for developing stable and high capacity all-solid-state Li ion batteries. At the present, the prototype cell using SnO_2_/C45[72] showed a specific energy density of 2040 Wh/kg at the specific power density of 268.6 W/kg based on the anode material weight which is even better performance than a liquid electrolyte-type cell. On the other hand, development of Si-, SiO_x_- and Ge-embedded porous carbons as a high capacity and stable electrode in OLE system was reported recently^20–22^. By optimization of the composite structure and exploiting the compositional versatility using Si, SiO_x_, Ge and so on, therefore, we could further extend the possible sets of electrodes for advanced all-solid-state batteries.

## Methods

### Synthesis of SnO_2_/nanoporous carbon composites

A nanoporous carbon with an average pore diameter (*X* nm) of 140 nm or 45 nm, which was denoted as C*X*, was obtained by a silica opal template process according to the previous report^19^. A mixture of SnCl_2_ (Kishida Chemical Co. Ltd.) and C*X* was heated in a sealed tube at 593 K for 24 h. The C*X* used was preheated at 393 K for 2 h under vacuum to remove adsorbed water. 400 mg of the mixed sample obtained above was dispersed in 600 mL of pure water and was filtered off to remove unreacted SnCl_2_, and then was dried in *vacuo* for overnight. The obtained composite is referred to as SnO_2_/C*X*[*Y*], where *Y* indicates wt% of the SnO_2_ content.

### Structural Characterization

Powder X-ray diffraction (XRD) patterns of samples were obtained on a Rigaku RINT-2200 diffractometer using CuKα radiation. The SnO_2_ content in samples was estimated by thermogravimetric analysis (TGA, SEIKO Instrument Inc, TG/DTA7300). The specific surface areas were determined by α_s_-plot analysis^23,24^ from the N_2_ adsorption isotherm at 77 K (MicrotracBEL Co. Ltd. BELSORP-max). Pore size distribution and pore volume of samples were analyzed by Grand Canonical Monte Carlo (GCMC) method^25^. The morphology of samples was observed by scanning electron microscope (SEM, JEOL JSM-7500FAM) and high-resolution scanning transmission electron microscope (HR-STEM; ARM-200CF, JEOL Ltd.). The SEM image and EDX analysis data of a cross-section of solid electrolyte/anode were taken on the material peeled off from the current collector sheet in an Ar-filled glove box after a half-cell construction described below.

### Electrochemical Measurements

A two electrode all-solid-state cell was used to investigate electrochemical properties of SnO_2_/C*X*[*Y*]. A glassy solid electrolyte (SE) was prepared by mechanical milling of a 10:90 mixture by weight% of LiI (Aldrich, 99.999%) and 75Li_2_S-25P_2_S_5_ (mol%) at 370 rpm for 40 hours with a planetary ball-milling apparatus (Fritsch Japan Co. Ltd, P-7), which was followed by heating at 583 K for 2 hours in vacuum. The 75Li_2_S-25P_2_S_5_ was obtained according to the previous paper^26^. For the working electrode, the SE and SnO_2_/C*X*[*Y*] with the volume ratio of 50:50 were dispersed in heptane by using a homogenizer, and then dried on a hot plate in an Ar atmosphere. A typical cell was fabricated in an Ar-filled glove box using a macol cylinder with 1.0 cm^2^ cross-section area as follows. The SnO_2_/C*X*[*Y*] and SE composite (16.8 mg) was pressed on a stainless-steel sheet at 1 ton/cm^2^ in the cylinder, and subsequently a SE layer (100 mg) was formed by pressing at 40 ton/cm^2^. Then a Li foil was placed on the surface of SE side of the bilayer to construct a half-cell. Cycling test was carried out by using a prototype full-cell which was fabricated by utilizing the SnO_2_/C*X*[*Y*] and SE composite as an anode and a 50:48:2 mixture by volume% of LiNbO_3_-coated LiNi_1/3_Co_1_/_3_Mn_1/3_O_2_ (Nb-LNCM), the SE and vapor grown carbon fiber (VGCF, Showa Denko K.K.) as a cathode. The Nb-LNCM was prepared according to the procedure previously reported^14^. The cell construction was carried out by the same manner as that for the half cell. The cathode and anode were assembled so that the capacity ratio of cathode/anode was 1.2.

Electrochemical charge-discharge curves were measured on an electrochemical analyzer (Hokuto Denko, HJ-SM8) at 25 °C. A charging-discharging at a constant current (CC) mode was carried out for the half-cell of ASS system at the current density of 0.1 C in the potential range of 0.01–2.5 V vs. Li/Li^+^ after the initial discharging from open circuit voltage to 0.01 V. The 0.1 C corresponds to the current density of 79 mA g^−1^ based on the weight of SnO_2_/C*X*[*Y*]. The full-cell test was performed by using a constant current and constant voltage (CC−CV) mode in the potential range of 4.5–2.0 V at the cut-off current 0.01 C. Electrochemical properties of SnO_2_/C*X*[*Y*] in an organic liquid electrolyte was also investigated according to the previous report^18^ using a Li counter electrode and a 1.0 mol dm^−3^ LiPF_6_ in ethylene carbonate/dimethyl carbonate (1/1 by volume).

## Electronic supplementary material

Supporting Information
