# Supplementary material for: High capacity and stable all-solid-state Li ion battery using SnO2-embedded nanoporous carbon
Source: Sci Rep. 2018 Jun 8;8:8747. doi: 10.1038/s41598-018-27040-w (PMC5993792; doi:10.1038/s41598-018-27040-w)
Supplement: Supplementary file 1 — Supporting Information [file 41598_2018_27040_MOESM1_ESM.pdf]

High capacity and stable all-solid-state Li ion battery using SnO_2-embedded nanoporous carbon

Hiroo Notohara,[1] Koki Urita,[1] Hideyuki Yamamura,[2] and Isamu Moriguchi[1*]

[1]Graduate School of Engineering, Nagasaki University, 1-14 Bunkyo-machi, Nagasaki-shi, Nagasaki 852-8521, Japan

[2]TOYOTA Motor Corporation, 1200 Mishuku, Susono-shi, Shizuoka 410-1193, Japan

Supporting Information

Contents

Figure 1. XRD patterns of SnO_2/C*X*[*Y*].

Figure 2. N_2 ad-/desorption isotherms and pore size distributions of SnO_2/C45[*Y*].

Figure 3. SEM image of as-prepared solid electrolyte.

Figure 4. STEM-EELS-EDX analyses of the interface between SnO_2/C140[62] and SE.

Figure 5. CV curves of SnO_2/C45[74] in ASS system measured at room temperature and scan rate of 0.2 mV/sec

Calculations

1. Theoretical volume ratio of the full expanded state against original SnO_2
2. Theoretical coverage ratio of SnO_2 against pore surface of CX

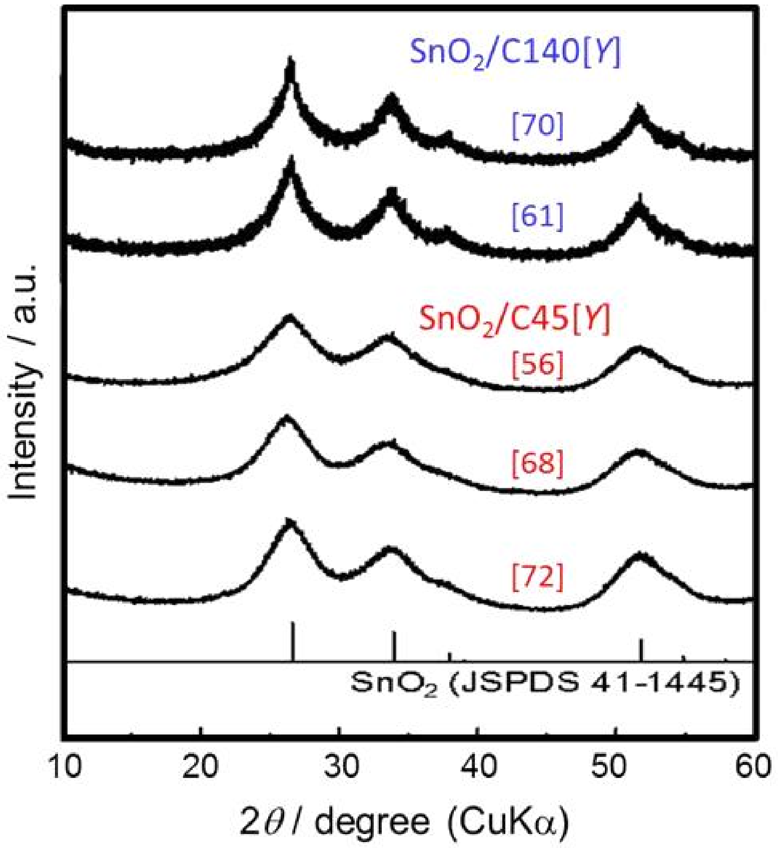

Supplementary Figure 1. XRD patterns of SnO_2/C*X*[*Y*].

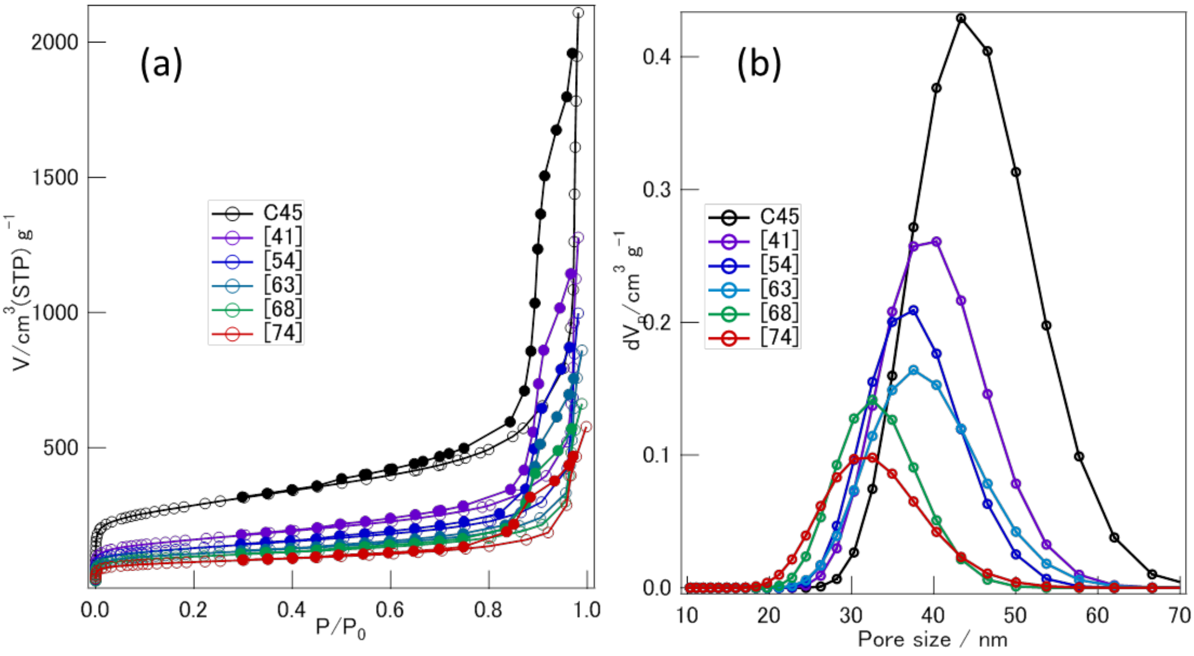

Supplementary Figure 2. N_2 ad-/desorption isotherms (a) and pore size distributions (b) of SnO_2/C45[*Y*].

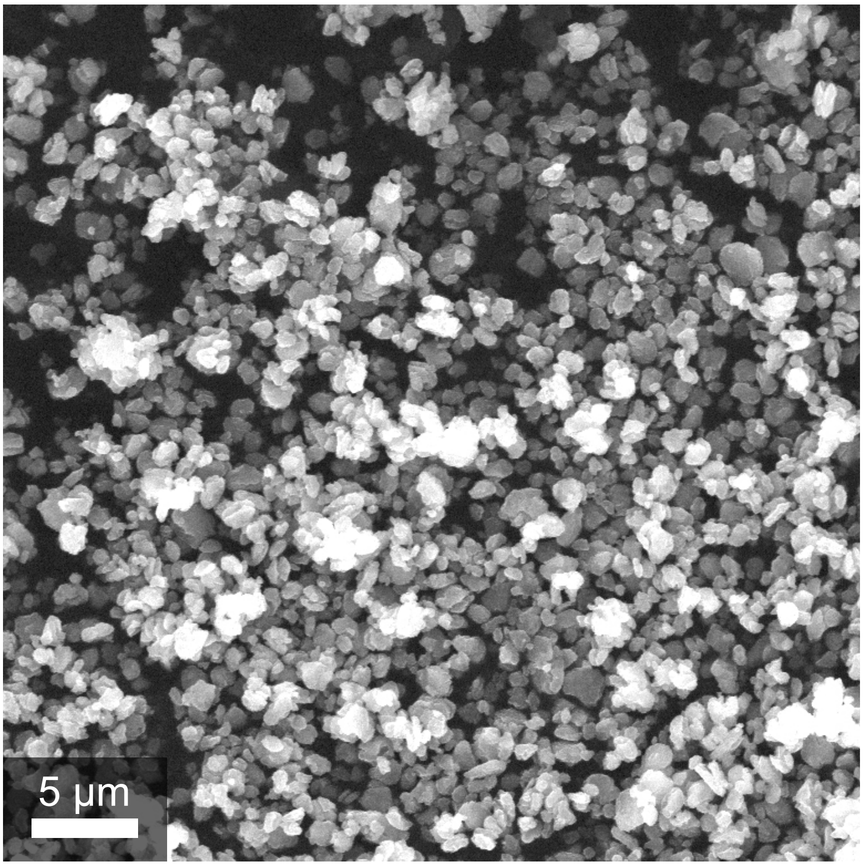

Supplementary Figure 3. SEM image of as-prepared solid electrolyte

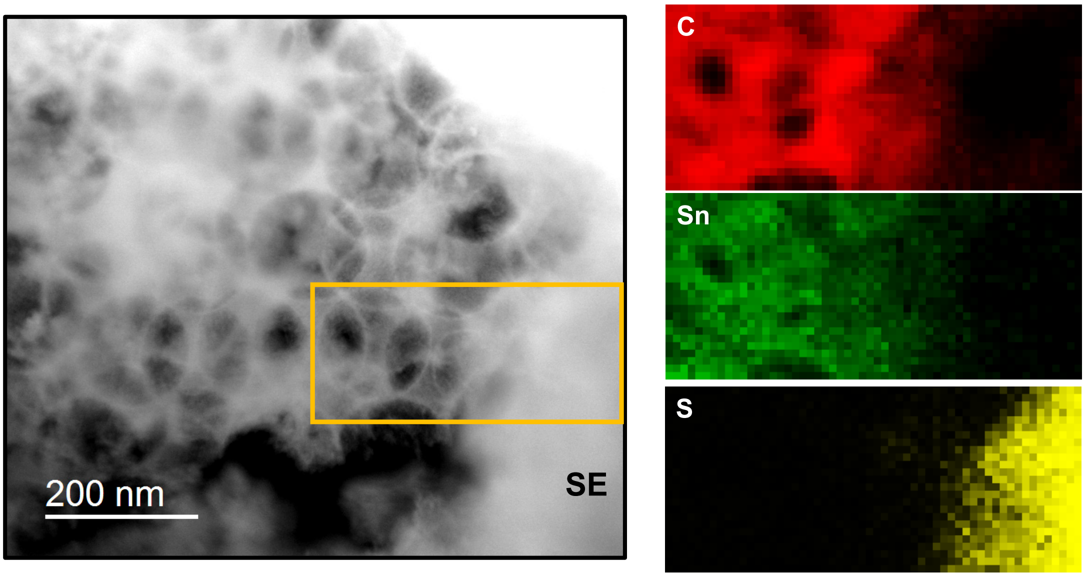

Figure 4. STEM-EELS-EDX analyses of the interface between SnO_2/C140[62] and SE. The analyses were performed on the sample directly put on a TEM grid after grinding the material peeled off from the current collector. Left: an annular dark field (ADF) image, Right: EELS maps of C and Sn, and an EDX map of S obtained for the rectangular areas depicted in the corresponding ADF images. These data were taken by a high-resolution scanning transmission electron microscope (HR-STEM; ARM-200CF, JEOL Ltd.) equipped with electron energy loss spectroscopy (EELS) and energy dispersive X-ray spectroscopy (EDX) at an accelerating voltage of 80 kV. EDX elementary analysis was adopted for S because EELS signal cannot be detected clearly in principle on a thick material such as SE part in the ADF image due to the multiple scattering of electron. No EELS signal of S in the porous part (SnO_2/C140[62]) was confirmed even by EELS.

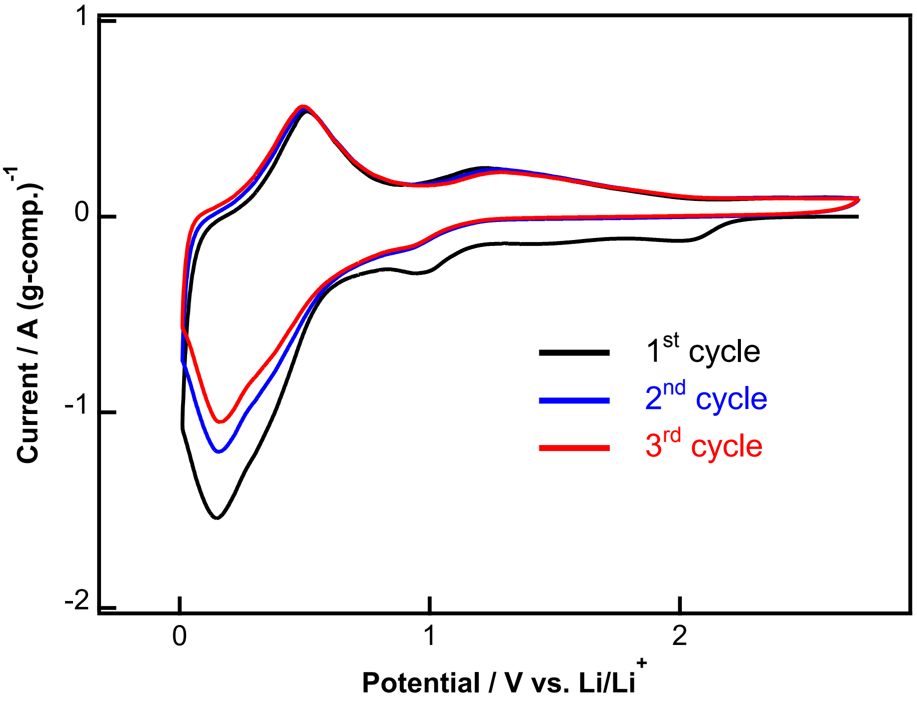

Figure 5. CV curves of SnO_2/C45[74] in ASS system measured at room temperature and scan rate of 0.2 mV/sec

Supplementary Calculations

1. Theoretical volume ratio of the full expanded state against original SnO_2

The theoretical volume ratio after the full reaction of SnO_2 with Li ions ([V_{expand}/V_{SnO2}]) was calculated to be 4.05 from the following equation (1):

$$\frac{V_{expand}}{V_{SnO_2}} = \frac{2\times (M_{Li_2O}/\rho_{Li_2O}) + (M_{Li4.4Sn}/\rho_{Li4.4Sn})}{(M_{SnO_2}/\rho_{SnO_2})} \qquad (1)$$

where M and ρ are molecular mass and density, respectively. The value of each material shown by subscript is as follows.

M_{SnO2} = 150.71 g mol^{-1}, ρ_{SnO2} =6.95 g cm^{-3} [1)]

M_{Li2O} = 29.88 g mol^{-1}, ρ_{Li2O} = 2.01 g cm^{-3} [2)]

$M_{Li4.4Sn}$ = 149.25 g mol^{-1}, $\rho_{Li4.4Sn}$ = 2.57 g cm^{-3} [3)]

References

1) *CRC Handbook of Chemistry and Physics*; Lide T. D. ed.; 75th edition; CRC press inc.; Boca Raton, 1994

2) Patnaik, P. *Handbook of inorganic chemicals*; McComb, K. ed.; The McGraw-Hill Companies, Inc.; New York, 2003

3) Gladyshevskii, E. I.; Oleksiv, G. I.; Kripyakevich, P. I. New Examples of The Structural Type $Li_{22}Pb_5$. *Sov. Phys. Crystallogr.* 1964, *9*, 269-271

2. Theoretical coverage ratio of SnO_2 against pore surface of C*X*

If spherical nanoparticles of SnO_2 with the diameter (d) of 3 nm are closely packed and form a single layer over the whole surface of mesopores and macropores of C*X*, the theoretical coverage ratio (θ) can be calculated by following equations (2) , (3) and (4):

$$\theta = 100\, S_{SnO_2}/S_c \qquad (2)$$

$$S_{SnO_2} = \frac{y}{\left(\frac{4\pi}{3}\right)\left(\frac{d}{2}\right)^3 \rho_{SnO_2}} \times 2\sqrt{3}\left(\frac{d}{2}\right)^2 \qquad (3)$$

$$Y = \frac{100\, y}{1 + y} \qquad (4)$$

where y is gram of SnO_2 per 1g of carbon in SnO_2/CX[Y] and d is particle diameter of SnO_2 (3 nm). S_{SnO_2} and S_c are the area of SnO_2 particulate monolayer and specific mesopore and macropore surface area of CX, respectively.

At the expansion state after the full reaction of SnO_2 with Li ions, the coverage ratio (θ_{expand}) can be express by equations (5) and (6):

$$\theta_{expand} = 100\, S_{expand}/S_c \qquad (5)$$

$$S_{expand} = S_{SnO_2} \times 4.05^{2/3} \qquad (6)$$

where S_{expand} is the area of SnO_2-related layer after the full reaction with Li ions.
